# Cross-Disorder Genomics Data Analysis Elucidates a Shared Genetic Basis Between Major Depression and Osteoarthritis Pain

**DOI:** 10.3389/fgene.2021.687687

**Published:** 2021-09-16

**Authors:** Sophie Barowsky, Jae-Yoon Jung, Nicholas Nesbit, Micah Silberstein, Maurizio Fava, Marco L. Loggia, Jordan W. Smoller, Phil H. Lee

**Affiliations:** ^1^Psychiatric and Neurodevelopmental Genetics Unit, Center for Genomic Medicine, Massachusetts General Hospital, Boston, MA, United States; ^2^Department of Pediatrics, Stanford University, Stanford, CA, United States; ^3^Department of Psychiatry, Harvard Medical School and Massachusetts General Hospital, Boston, MA, United States; ^4^Stanley Center for Psychiatric Research, Broad Institute of MIT and Harvard, Cambridge, MA, United States; ^5^Depression Clinical and Research Program, Department of Psychiatry, Massachusetts General Hospital, Boston, MA, United States; ^6^Athinoula A. Martinos Center for Biomedical Imaging, Massachusetts General Hospital, Boston, MA, United States

**Keywords:** pleiotropy, major depression, osteoarthritis, pain, mechanosensory behavior, cross-disorder GWAS, comorbidity

## Abstract

Osteoarthritis (OA) and major depression (MD) are two debilitating disorders that frequently co-occur and affect millions of the elderly each year. Despite the greater symptom severity, poorer clinical outcomes, and increased mortality of the comorbid conditions, we have a limited understanding of their etiologic relationships. In this study, we conducted the first cross-disorder investigations of OA and MD, using genome-wide association data representing over 247K cases and 475K controls. Along with significant positive genome-wide genetic correlations (*r*_g_ = 0.299 ± 0.026, *p* = 9.10 × 10^–31^), Mendelian randomization (MR) analysis identified a bidirectional causal effect between OA and MD (β_OA__→__MD_ = 0.09, SE = 0.02, *z*-score *p*-value < 1.02 × 10^–5^; β_MD__→__OA_ = 0.19, SE = 0.026, *p* < 2.67 × 10^–13^), indicating genetic variants affecting OA risk are, in part, shared with those influencing MD risk. Cross-disorder meta-analysis of OA and MD identified 56 genomic risk loci (*P*_meta_ ≤ 5 × 10^–8^), which show heightened expression of the associated genes in the brain and pituitary. Gene-set enrichment analysis highlighted “*mechanosensory behavior*” genes (GO:0007638; *P*_gene_set_ = 2.45 × 10^–8^) as potential biological mechanisms that simultaneously increase susceptibility to these mental and physical health conditions. Taken together, these findings show that OA and MD share common genetic risk mechanisms, one of which centers on the neural response to the sensation of mechanical stimulus. Further investigation is warranted to elaborate the etiologic mechanisms of the pleiotropic risk genes, as well as to develop early intervention and integrative clinical care of these serious conditions that disproportionally affect the aging population.

## Introduction

Osteoarthritis (OA) is the most frequent form of arthritis, affecting over 32.5 million adults in the United States [Centers for Disease Control and Prevention (CDC), 2020]. The cardinal symptom of OA is debilitating and chronic pain that affects synovial joints, including the knees, hips, and back, often aggravated by stiffness, swelling, and reduced mobility. Patients with OA often struggle with major depression (MD) and anxiety, linked with greater burden of pain severity, functional disability, and increased mortality (Fuller-Thomson et al., 2016; Sharma et al., 2016; Sambamoorthi et al., 2017); a longitudinal study using the United States National Institute of Health OA Initiative data has reported higher odds of developing depressive symptoms in individuals with hip, knee, or multi-site OA [Odds Ratio (OR): 1.43 ∼ 1.72] (Veronese et al., 2016). Similarly, Akintayo et al. (2019) reported that 42% of OA patients suffer from MD (OR: 2.49).

**FIGURE 1:**
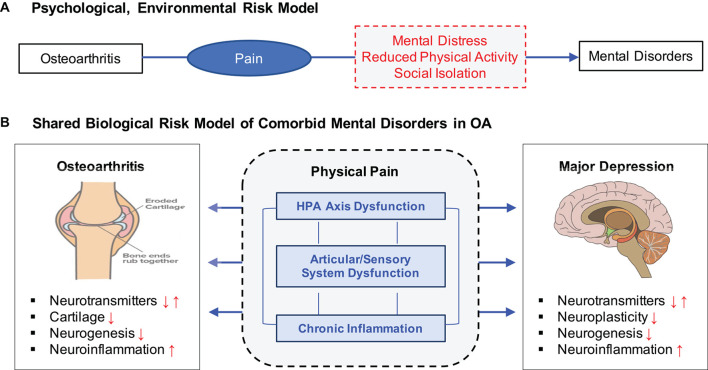
Two distinct but not mutually exclusive risk models for OA, pain, and MD. **(A)** Psychological, environmental risk model attributes various negative health outcomes of OA as a major contributing factor to increasing risk to MD. **(B)** Shared biological risk model proposes that OA and MD are affected by some common biological mechanisms related to stress response, chronic inflammation, and articular/sensory neuronal dysfunction.

The higher comorbidity of MD and OA has been interpreted in two major ways (Figure 1). Under the psychological, environmental risk model, chronic pain has been identified as a major mediating risk factor leading to the increased risk of depression in OA patients (Mammen and Faulkner, 2013; Veronese et al., 2016). Reduced physical activity and few social interactions are also common in people suffering from OA (Wallis et al., 2013) and especially those affected in the lower parts of the body such as the knee and hip (Stubbs et al., 2015). In contrast, clinical studies have suggested that OA and MD may share some common etiologic mechanisms related to stress, inflammation, and immune responses. MD has been associated with increased inflammatory activation of the immune systems in both the periphery and the central nervous systems (Lee and Giuliani, 2019), while OA patients exhibit higher inflammatory markers (Bonnet and Walsh, 2004) and cortisol levels (Carlesso et al., 2016), indicating altered immune systems. Neurobiologic studies have indicated that the autonomic nervous system has a role in joint homeostasis and OA pathogenesis, suggesting pain sensitization (Clauw and Hassett, 2017; Syx et al., 2018) and neuronal impairment (McDougall, 2019) as potential risk factors connecting OA and MD.

In this study, we examined the etiologic relationships between OA, pain, and MD using cross-disorder genome-wide genetic data analysis. Specifically, we aimed to clarify whether OA and MD share common genetic risk mechanisms that increase susceptibility to both conditions (known as biological pleiotropy), or genetic risk influencing OA and pain mediates increasing risk to MD (known as mediated pleiotropy). To this aim, we applied a series of pleiotropy analyses, using by far the largest genome-wide genetic variation data of the two disorders, representing a sample of 77 K OA cases, 171 K MD cases, and 450 K controls. First, we measured genome-wide genetic correlations between OA and MD and inspect their potential causal relationships using Mendelian randomization (MR) analyses. Further, we performed a cross-disorder genome-wide association study (GWAS) to identify specific risk loci, genes, and biologic mechanisms that may influence the two health conditions. Lastly, we investigated five drug-gene interaction databases to clarify clinical implications of the shared etiology. To the best of our knowledge, this is the first investigation of this kind.

## Materials and Methods

### GWAS Datasets

We obtained the latest GWAS summary statistics for OA (Tachmazidou et al., 2019), which investigated a total of 455,221 individuals (77,052 OA cases and 378,169 controls). We obtained GWAS summary statistics for MD from the Psychiatric Genomics Consortium (PGC) (Howard et al., 2019). This study was based on a total of 170,756 MD cases and 329,443 controls. Table 1 summarizes the statistics of the two GWAS datasets. For further details, we refer to the original publications (Howard et al., 2019; Tachmazidou et al., 2019).

**TABLE 1:** Summary of GWAS datasets.

Trait	# Cases	# Controls	# Total sample	Population prevalence	Sample prevalence	Reference (PMID)
Major depression	170,756	329,443	500,199	0.15	0.341	Howard et al., 2019 (PMID: 30718901)
Osteoarthritis	77,052	378,169	455,221	0.13	0.169	Tachmazidou et al., 2019 (PMID: 30664745)

*Number of cases and controls, total sample size, population and sample prevalence, and PubMed ID for two GWAS for major depression (MD) and osteoarthritis (OA).*

### Quality Control and Standardization of GWAS Datasets

We applied unified quality control (QC) of the two GWAS summary statistics. Original datasets included 8.91 M SNPs for OA and 8.48 M SNPs for MD. First, chromosome coordinates were standardized to the reference genome GRCh37/hg19. We removed SNPs that were rare (minor allele frequency < 1%), non-biallelic, lack of rs IDs, not present in the 1,000 Genomes Project Phase 3 European samples, and had incompatible alleles. We also removed palindromic SNPs, with allele frequency differences less than 15%. After QC, 7.85 M and 7.75 M SNPs retained for OA and MD, respectively. We restricted analysis to autosomal chromosomes.

### Estimation of Polygenicity, Discoverability, and Genetic Correlation

To compare the genetic architecture of MD and OA, we estimated the polygenicity (defined as the fraction of independent causal SNPs, or π) and discoverability (the variance of causal SNP effect sizes, or $\sigma_{\beta }^{2}$) using a univariate Gaussian mixture model implemented in MiXeR (Holland et al., 2020). Genome-wide genetic correlation was estimated using LDSC (linkage disequilibrium score regression) with pre-computed European LD scores (Bulik-Sullivan et al., 2015). For measuring a liability-based SNP heritability, we used a population prevalence compiled from the literature: 13.46% for OA (Tachmazidou et al., 2019) and 15% for MD (Lee et al., 2013). We used the GCTA-GREML power calculator (Visscher et al., 2014) to calculate the statistical power of estimating genetic correlation of at least 0.2 between OA and MD with default setting (α = 0.05). We ran the sign-concordance test between OA and MD using the binomial test in R, assessing how often genome-wide significant SNPs for one phenotype show the same directional effect with the other phenotype. We expect sign concordance of 50% between any random phenotypes, and the estimated binomial probability represents how unlikely it is to observe the assessed concordance between two phenotypes by chance.

### Mendelian Randomization

Mendelian randomization examines whether relationships between an exposure and an outcome are causal (Minca et al., 2020). We performed bi-directional, two-sample MR analysis between OA and MD using GSMR (Generalized Summary-data-based Mendelian Randomization) (Zhu et al., 2018). This multi-step method uses genome-wide significant SNPs for individual traits (p ≤ 5 × 10^–8^) as instrumental variables in each unidirectional test. GSMR takes into account linkage disequilibrium (LD) between genetic variants and sample variance in genetic effects on exposure and outcome for each SNP. We estimate the causal effect (${\hat{b}}_{x\,y}$) of the exposure (${\hat{b}}_{z\,x}$) on the outcome (${\hat{b}}_{z\,y}$) as:


$${\hat{b}}_{x\,y}={\hat{b}}_{z\,y}/{\hat{b}}_{z\,x}$$

We used default settings for the method with the 1,000 genomes-based European samples for estimating LD. To avoid potential bias and test-statistic inflation due to pleiotropy, we used the HEIDI-outlier (heterogeneity in dependent instruments) method to detect putative pleiotropic SNPs.

### Cross-Disorder Meta-Analysis

We conducted meta-analysis of OA and MD using METAL (Willer et al., 2010). This method adjusts potential sample overlaps by first estimating the covariance of the *Z*-score distribution and assigning proper weights while summing *Z*-scores. Optimal weights were previously represented by Lin and Sullivan’s work (Lin and Sullivan, 2009):


$$\left[w_{1},\ldots \,w_{k}\right]=e^{T}\,\Omega^{-1}/e^{T}\,\Omega^{-1}\,e$$

where *w*_*k*_ is the weight for the *k^*th*^* study, *e* is a *K*×1 vector of 1’s and Ω is the estimated covariance matrix of (*Z*_1_,…*Z*_*k*_). To estimate the covariance matrix, *Z*-scores were truncated to remove non-null effects using the absolute *Z*-score threshold of 1. We then meta-analyzed the two datasets using:


$$\hat{Z}=\frac{1}{\sqrt{\sum_{k}w_{k}^{2}+\sum_{k}\sum_{l\neq k}w_{k}\,w_{l}\,{\hat{r}}_{k\,l}}}\,\sum_{k=1}^{K}w_{k}\,Z_{k}$$

where ${\hat{r}}_{k\,l}$ is the estimated correlation between *Z*-scores of the *k^*th*^* and *l^*th*^* studies under the null. A total of 7,433,563 SNPs were meta-analyzed. We used LDSC and genomic inflation factors to assess inflation due to potential confounding factors of the meta-analysis results (Clayton et al., 2005).

### Functional Annotation of Risk Loci Using FUMA

With summary-level association statistic results from cross-disorder meta-analysis as input, we performed functional annotation of risk loci using FUMA (Watanabe et al., 2017). We used default settings, while excluding the MHC region (chr6:25–35 Mb) for annotations and gene-set enrichment analysis. Gene mapping of SNPs was based on functional consequences on genes from positional, eQTL, and chromatin interaction mapping data in the brain. For annotations, we included protein coding and non-coding RNAs.

### Gene-Based Association Test and Gene-Set Enrichment Analysis

Gene-set enrichment analysis was performed using MAGMA v1.6 (20 kb gene window) (de Leeuw et al., 2015) and the MSigDB v7.0 Gene Ontology (GO) data (Liberzon et al., 2011). We also ran gene-level analysis using cross-disorder meta-analysis summary statistics. Significant genes were designated using a *p*-value threshold *P*_meta_ ≤ 2.64 × 10^–6^ (=0.05/18,939 genes) for adjusting multiple testing of 18,939 genes at alpha = 0.05. Among the identified genes with significant association, we prioritized the ones that show suggestive association with both OA and MD (*P*_OA_ ≤ 1 × 10^–4^, *P*_MD_ ≤ 1 × 10^–4^). Tissue-specific gene expression analysis was conducted with GTEX data (v8) (GTEx Consortium, 2020).

### Regional Brain and Cell-Type-Specific Expression Analysis

We examined whether shared OA/MD risk genes show cell-type specific gene expression using six brain cell type RNA sequencing data: astrocyte, endothelial, microglia, neuron, oligodendrocyte, and OPC (Darmanis et al., 2015). To designate significant RNA expression for each cell type, we used two criteria suggested in the original publication: mean expression threshold of at least five and a *Z*-score of 2. We also investigated spatiotemporal gene expression using the Human Brain Transcriptome (Kang et al., 2011).

### Search of Drug-Gene Interaction Databases

We investigated drug-gene interactions related to OA and MD using five databases: DGIdb v3.0 (Cotto et al., 2017), DrugBank v5.0 (Wishart et al., 2017), PharmGKB (Whirl-Carrillo et al., 2012), STITCH v5.0 (Szklarczyk et al., 2015), and TTD 2020 (Wang et al., 2019). First, we compiled a list of OA- and MD-associated genes identified from disease-specific gene-based association analyses, shared OA/MD risk genes obtained from cross-disorder meta-analysis, and member genes of the gene-sets for which we identified significant cross-phenotype association in gene-set enrichment analyses described in the previous sections. Additionally, we included a list of OA and MD-associated genes compiled from the original GWAS studies (Howard et al., 2019; Tachmazidou et al., 2019).

## Results

### Osteoarthritis and Major Depression Share Significant Genome-Wide Genetic Correlations

We have assembled GWAS datasets of OA (*N* = 77,052 cases and 378,169 controls) and MD (*N* = 170,756 cases and 329,443 controls), which represent by far the largest publicly available studies of the two disorders (Table 1). Statistical power analysis indicated >99% power for detecting SNP-based heritability and genetic correlation in our datasets (Supplementary Table 1). Significant SNP-based heritability was estimated for both disorders on the liability scale: OA (*h*_2_ = 9.4%, SE = 0.004) and MD (*h*_2_ = 8.5%, SE = 0.003). The disorders were also highly polygenic, involving thousands of causal risk variants; approximately 8.7 K variants were estimated to causally influence OA and 13.7 K influence MD. Sign tests of genome-wide significant SNPs indicated that 67 out of 90 genome-wide significant SNPs for MD share the same directional effect with OA (binomial sign test *P*_MD_→_OA_ = 3.80 × 10^–6^), while 33 out of 51 OA index SNPs share direction with MD (*P*_OA_→_MD_ = 4.89 × 10^–2^). We also identified a substantial and statistically significant level of positive genome-wide genetic correlation between the two disorders (*r*_g_ = 0.30 ± 0.026, *p* = 9.10 × 10^–31^).

### Mendelian Randomization Predicts Shared Genetic Etiology Between OA and MD

**FIGURE 2:**
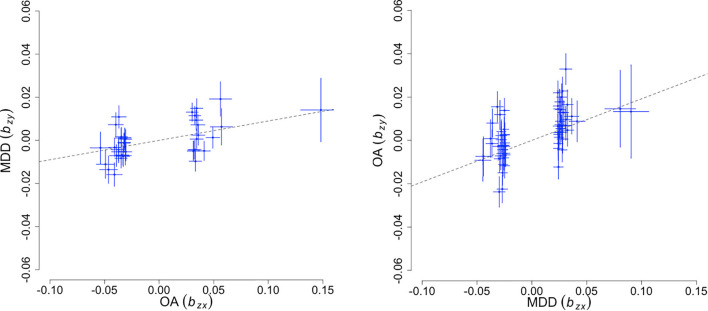
Bidirectional Mendelian randomization analysis results. The X-axis represents the estimated effect of the SNPs associated with the exposure (${\hat{b}}_{z\,x}$), while the Y-axis represents the estimated effect of the same SNPs on outcome (${\hat{b}}_{z\,y}$). Each cross marks independent instruments (i.e., SNPs) used to test for causality. The ratio, ${\hat{b}}_{z\,y}$/${\hat{b}}_{z\,x}$ is an estimate of the mediation effect of the exposure on the outcome assuming that under a causal model, if an exposure has a causal effect on an outcome, all instruments causally associated with the exposure will have an effect on the outcome expected to be identical.

To infer potential causality in the relationship between OA and MD, we performed bi-directional MR analyses using a GSMR method. As input, we used 39 and 64 linkage disequilibrium (LD)-independent SNPs that show genome-wide significant association with OA and MD, respectively. We ran GSMR under a default-setting while removing SNPs displaying horizontal pleiotropy (HEIDI outlier *p*-value < 0.01). Figure 2 illustrates the bidirectional MR analysis results. When using MD as the risk factor and OA as the outcome, we found an effect size β_MD__→__OA_ of 0.192 (SE = 0.026, *p* < 2.67 × 10^–13^). The reverse-directional MR test estimated a significant but more modest effect of OA on MD (β_OA__→__MD_ = 0.09 ± 0.02, *p* < 1.02 × 10^–5^).

### Cross Disorder Meta-Analysis Highlights a Role of Mechanosensory Behaviors

**FIGURE 3:**
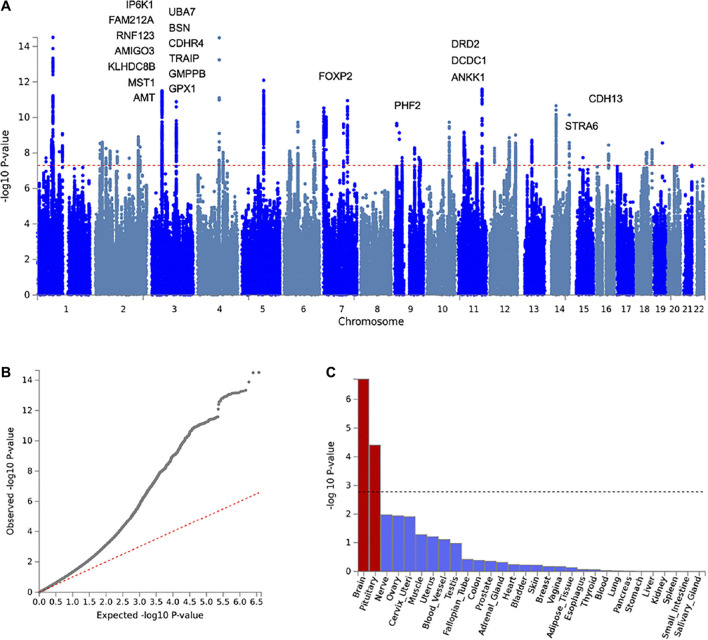
Cross-disorder meta-analysis results of OA and MD. **(A)** Manhattan plot with labels for the top 20 of 42 genes significantly associated with both OA and MD (gene-based *P*_meta_ ≤ 2.64 × 10^–6^, *P*_OA_ ≤ 1 × 10^–4^, *P*_MD_ ≤ 1 × 10^–4^). **(B)** Q-Q plot of the meta-analysis results showing expected vs. observed –log(*P*) for examined SNPs. The red dotted line indicates a situation when observed *p*-values exactly match what would be expected by random chance. **(C)** Graphic showing results of tissue-specific enrichment analysis for 30 general tissue types. The X-axis represents the examined tissues, while the Y-axis represents the enrichment *p*-values at the minus log scale. The black dotted line indicates a statistical significance level after multiple testing corrections.

To elucidate shared genetic risk mechanisms, we conducted a cross-disorder meta-analysis of OA and MD. Figure 3 summarizes the genome-wide meta-analysis results of 7.43 M SNPs. LDSC analysis indicates that the observed inflation in the QQ-plot is due to underlying polygenic signals of the two disorders rather than confounding factors (LDSC intercept = 0.9816 ± 0.01). Genomic inflation factor λ_1,000_ was 1.001. After LD-based pruning, we identified 56 genomic risk loci represented by 63 lead SNPs (SNP-based association *P*_meta_ ≤ 5 × 10^–8^; Supplementary Table 2). The identified loci were annotated using various functional genomics data using FUMA, including eQTL, Hi-C, and positional mapping (Supplementary Table 3). These genes showed heightened expression in the brain (enrichment β = 0.044, SE = 0.009, *p* = 1.98 × 10^–7^) and pituitary tissues (β = 0.041, SE = 0.010, *p* = 3.94 × 10^–5^) (Supplementary Tables 4, 5).

**FIGURE 4:**
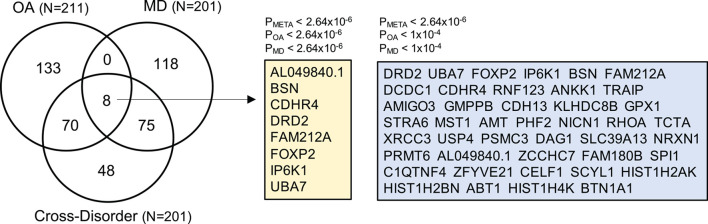
Venn diagram summarizing the overlap of gene-based analysis results from OA, MD, and cross-disorder GWAS. The numbers in the Venn diagram represent the number of shared and/or distinct genes identified as statistically significant in three gene-based association analyses after Bonferroni correction (*p* < 2.64e-06). The total number of OA-, MD-, and cross-disorder associated genes are listed in the parentheses. There are eight genes for which all three gene-based analysis showed genome-wide significant association (listed in yellow box). In the right blue box, we list 43 genes, which showed genome-wide significant association in meta-analysis-based gene association analysis, and suggestive association at *p* < 1e-04 in OA- and MD-specific association.

To prioritize specific genes that could increase susceptibility to both OA and MD, we ran gene-based association analysis using MAGMA (see section “Materials and Methods”). We identified 201 statistically significant genes after Bonferroni correction (gene-based *P*_meta_ ≤ 2.64 × 10^–6^). Of those, 43 genes showed suggestive association with both OA and MD, individually (gene-based *P*_OA_ ≤ 1 × 10^–4^, *P*_MD_ ≤ 1 × 10^–4^). We summarized the top 20 risk genes in Table 2 (full list in Supplementary Table 6; Figure 4). The top five genes based on statistical significance included *DRD2* (*P*_meta_ = 1.78 × 10^–15^), *UBA7* (*P*_meta_ = 8.45 × 10^–12^), *FOXP2* (*P*_meta_ = 1.07 × 10^–11^), *IP6K1* (*P*_meta_ = 2.01 × 10^–11^), and *BSN* (*P*_meta_ = 2.73 × 10^–11^).

**TABLE 2:** List of top 20 pleiotropic OA/MD risk genes.

Gene	Locus	Name	*P*-value OA	*P*-value MD	*P*-value meta-analysis
DCDC1	p13	Doublecortin domain containing 1	2.35e-08	5.00e-05	3.13e-11
AMIGO3	p21.31	Adhesion molecule with Ig-like domain 3	2.18e-09	1.06e-05	9.91e-11
AMT	p21.31	Aminomethyltransferase	4.91e-07	4.40e-06	2.04e-09
BSN	p21.31	Bassoon presynaptic cytomatrix protein	3.13e-08	2.52e-07	2.73e-11
CDHR4	p21.31	Cadherin-related family member 4	1.35e-08	2.95e-07	3.20e-11
FAM212A	p21.31	Family with sequence similarity 212, member A	1.12e-08	4.41e-07	2.91e-11
GMPPB	p21.31	GDP-mannose pyrophosphorylase B	2.18e-09	1.06e-05	9.91e-11
GPX1	p21.31	Glutathione peroxidase 1	3.42e-07	6.22e-06	1.18e-09
IP6K1	p21.31	Inositol hexakisphosphate kinase 1	1.21e-09	2.08e-06	2.01e-11
KLHDC8B	p21.31	Kelch domain containing 8B	9.42e-05	1.21e-07	1.03e-09
MST1	p21.31	Macrophage stimulating 1 (hepatocyte growth factor-like)	4.08e-08	5.68e-05	1.62e-09
RNF123	p21.31	Ring finger protein 123	1.14e-09	4.82e-06	3.25e-11
TRAIP	p21.31	TRAF interacting protein	3.35e-10	1.40e-05	3.87e-11
UBA7	p21.31	Ubiquitin-like modifier activating enzyme 7	2.44e-09	2.57e-07	8.45e-12
PHF2	q22.31	PHD finger protein 2	2.57e-05	4.04e-06	2.36e-09
ANKK1	q23.2	Ankyrin repeat and kinase domain containing 1	2.02e-05	6.35e-09	3.83e-11
DRD2	q23.2	Dopamine receptor D2	1.16e-06	3.03e-14	1.78e-15
CDH13	q23.3	Cadherin 13	2.37e-05	2.94e-08	1.19e-10
STRA6	q24.1	Stimulated by retinoic acid 6	2.96e-07	5.35e-05	1.20e-09
FOXP2	q31.1	Forkhead box P2	1.14e-07	1.96e-06	1.07e-11

*Top 20 shared OA/MD risk genes out of 42 statistically significant shared risk genes (gene-based *P*_meta_ ≤ 2.64 × 10^–6^, *P*_OA_ ≤ 1 × 10^–4^, *P*_MD_ ≤ 1 × 10^–4^). Table includes gene symbol, locus, full name, and *p*-values for individual analyses (OA, MD) and meta-analysis.*

We examined cell type-specific gene expression of the 43 OA/MD risk genes. More than a half of the OA/MD risk genes showed highly enriched expression in neurons (24 out of 43 genes), followed by astrocytes (nine out of 43) (Supplementary Table 7). Many genes showed significant expression in all six brain regions we assessed (cerebellar cortex, mediodorsal nucleus of the thalamus, striatum, amygdala, hippocampus, and neocortex) (Supplementary Table 8). More than three quarters of the brain-expressed genes were also significantly expressed both pre- and postnatally, suggesting their significant role throughout the lifespan.

Gene-set enrichment analysis was conducted using GO data to prioritize specific biological mechanisms shared between OA and MD (Supplementary Table 9). Only the “*mechanosensory behavior*” gene set retained statistical significance after applying multiple testing correction (GO:0007638; β = 1.43 ± 0.26, *P*_meta_ = 2.45 × 10^–8^, *P*_bon_ = 2.54 × 10^–4^). Importantly, this gene set showed statistically significant association with both MD and OA, individually (*P*_MD_ = 2.48 × 10^–6^, *P*_OA_ = 1.76 × 10^–5^). The “*mechanosensory behavior*” gene-set contains 18 genes, of which 15 genes were covered by our GWAS data. Several risk genes we identified from cross-disorder meta-analysis also belong to this gene set, including *DRD2, FOXP2, NRXN1*, and *STRA6*.

### Investigation of Drug-Gene Interactions Suggests Additional Genetic Ties

To investigate potential antagonistic or shared drug-gene interactions related to OA and MD, we investigated five drug-gene databases: DGIdb (Cotto et al., 2017), DrugBank (Wishart et al., 2017), PharmGKB (Whirl-Carrillo et al., 2012), STITCH (Szklarczyk et al., 2015), and Therapeutic Target Database (TTD) (Wang et al., 2019). The list of genes we investigated included 42 OA/MD shared risk genes, 195 that carry specific risk to OA, 179 MD-specific risk genes, and 18 “mechanosensory behavior” genes. Some genes fit into more than one category, resulting in a total of 430 genes (Supplementary Table 10). The analysis revealed 16 genes with drug-gene interactions of relation to OA and MD (Table 3 and Supplementary Table 11). There were seven OA-specific risk genes that we found interacting with psychiatric medications, including antidepressants: *CRHR1* (number of implicated medications *N* = 19), *CYP1A2* (*N* = 4), *IL11* (*N* = 2), *ITIH3* (*N* = 1), *TGFB1* (*N* = 1), *DPEP1* (*N* = 1), and *SLC39A8* (*N* = 1). Among 35 MD-associated genes with drug-interaction data, four MD-specific genes interacted with drugs associated with arthritis: *NR3C1* (*N* = 6), *NRG1* (*N* = 2), *CD40* (*N* = 2), *TYR* (*N* = 1). These drugs were classified as analgesics, anesthetics, and glucocorticoids (e.g., dexamethasone and triamcinolone). Three “mechanosensory behavior” genes, *DRD2*, *HTT*, and *NRXN1*, also featured multiple drug-gene interaction data (Table 3).

**TABLE 3:** Drug-gene interactions for OA/MD risk and “mechanosensory behavior” genes.

Gene	Category	Database	Drugs	Associated conditions
CD40	MD	TTD	ANTI-CD40-XTEN, BI 655064, CFZ533	Rheumatoid arthritis, myasthenia gravis
GRM5	MD	DrugBank, TTD	AZD2066, AZD2516, LY467711, LY525327, Rufinamide	Chronic neuropathic pain, migraine
NR3C1	MD	DGIdb, DrugBank, TTD	AZD9567, Dexamethasone, NCX-1015, PF-251802, Triamcinolone	Post-traumatic OA, gouty arthritis, rheumatoid arthritis, psoriatic arthritis
NRG1	MD	TTD	Aspirin, Bupivacaine, Colchicine	OA, acute gouty arthritis, psoriatic arthritis, rheumatoid arthritis
TAOK3	MD	PharmGKB	Morphine, opioids	Pain
TYR	MD	TTD	Rosmarinic acid	Rheumatoid arthritis
HTT	Mechano-sensory	DGIdb, PharmGKB, TTD	Risperidone, methylphenidate	Schizophrenia, ASD, bipolar I disorder, MDD, PTSD, alcoholism, anorexia nervosa, anxiety, OCD, ADHD, narcolepsy
CRHR1	OA	DGIdb, TTD, PharmGKB, STITCH	Antalarmin, Emicerfont, Fluoxetine, Pexacerfont, Telavancin	MDD, drug addiction, anxiety disorder, alcoholism, mood disorder
CYP1A2	OA	PharmGKB	Antipsychotics, Clozapine, Escitalopram, Paroxetine	Schizophrenia, MDD, anxiety, OCD, PTSD, panic disorders
DPEP1	OA	STITCH	Glutamic acid	Schizophrenia, alcoholism
IL11	OA	PharmGKB, DGIdb	Citalopram, Escitalopram	MDD, anxiety, OCD
ITIH3	OA	PharmGKB	Clozapine	Schizophrenia, ASD, bipolar I disorder, MDD, PTSD, anxiety, ADHD
SLC39A8	OA	PharmGKB	Ethanol	Alcoholism
TGFB1	OA	DGIdb	Atenolol	Alcohol withdrawal, migraine
NRXN1	Shared, mechano-sensory	DrugBank, PharmGKB	Antipsychotics, Duloxetine	MDD, anxiety, schizophrenia, fibromyalgia, musculoskeletal pain, knee OA, chronic lower back pain
DRD2	Shared, OA, MD, mechano-sensory	DGIdb, DrugBank, STITCH, TTD	Aripiprazole, Bicifadine, Clozapine, Lamotrigine, Risperidone	MDD, suicidal behavior, anxiety, PTSD, OCD, schizophrenia, alcohol withdrawal, ASD, ADHD, Parkinson’s disease, clinical trials for OA, migraine, neuropathic pain

*Drug-gene interaction information for 16 genes classified as OA, MD, shared, and mechanosensory. Table includes database sources, up to five relevant drugs, and associated conditions. For more associated conditions and full list of drugs, see Supplementary Table 11.*

## Discussion

While estimated prevalence varies depending on the sample size, population, and measurement tools, epidemiology studies have consistently reported the increased comorbidity between OA and MD (Gandhi et al., 2015; Hooten, 2016; Tsuji et al., 2019). The health burden of comorbid OA and MD is surprisingly harmful, spanning from greater symptom severity (de Heer et al., 2014) to poorer treatment outcomes (Roughan et al., 2020) to increased suicide attempts (Fuller-Thomson et al., 2016). Using the GWAS datasets of over 697 K individuals, our study shows for the first time that the increased comorbidity of OA and MD is, at least in part, due to a shared genetic etiology.

Cross-disorder meta-analysis of OA and MD revealed important insights into potential genetic mechanisms shared between the two conditions. First, we identified 63 LD-independent genome-wide significant SNPs associated with OA and MD. Implicated risk genes showed heightened expression in the brain and pituitary but not in other tissue types, suggesting that the shared genetic mechanisms between the two conditions are involved in the central nervous system and the endocrine system. Specific to the brain, we found significant enrichment of the shared risk genes in multiple brain regions, including the frontal cortex, the anterior cingulate cortex (ACC), and amygdala. Neuroimaging studies have reported a number of brain regions that play an important role in depression and pain perception, many of which converge to the ACC and amygdala (Sellmeijer et al., 2018; Serafini et al., 2020). In fact, Cottam et al. (2016) found that OA pain intensity correlates with blood flow in brain regions including the ACC and amygdala. Similarly, Brown et al. (2020) observed altered amygdala connection density in depressed patients. Our findings support the implication of the ACC and amygdala, along with other brain regions, in the shared genetic basis connecting OA, pain, and MD.

Secondly, our analysis revealed for the first time a statistically significant association of “mechanosensory behavior” genes with OA and MD. This gene set comprises 18 genes, and our GWAS data had coverage of 15 genes. Mechanosensory behavior genes are involved in mechanical stimuli, such as emotional processes and behaviors related to pain and mechano-sensation. Interestingly, autism spectrum disorder (ASD) mouse models with dysfunctional peripheral mechanosensory neurons have demonstrated touch hypersensitivity and altered tactile discrimination, contributing to difficulties in social interaction and anxiety symptoms (Orefice et al., 2016). Mechanosensation may also play a role in exacerbating pain through chronic stress, increasing synaptic efficiency in the amygdala, which combines nociceptive and affective information (Li et al., 2017). Additionally, certain mechanosensory nerves have been found to trigger the release of oxytocin in response to low intensity cutaneous stimulation and may serve to reward physical contact with others, reduce physiological arousal, and inhibit pain (Fingleton et al., 2015; Clauw and Hassett, 2017; Walker et al., 2017).

It is notable that the “mechanosensory behavior” genes not only showed significant enrichment *en masse*, but also included five genome-wide significant genes in cross-disorder meta-analysis (*DRD2*, *NRXN1*, *HTT*, *FOXP2*, and *STRA6*). Of these genes, *DRD2* (dopamine receptor D2) featured the most significant cross-disorder gene-based association (*P*_meta_ = 1.78 × 10^–15^, *P*_OA_ = 1.16 × 10^–6^, *P*_MD_ = 3.03 × 10^–14^). This gene encodes the D2 subtype of the dopamine receptor, a G-protein coupled receptor that inhibits adenylyl cyclase activity and plays a major role in reward, learning, and memory processes (Richter et al., 2017; Yin et al., 2020). *DRD2* is a well-established psychiatric risk gene, and has shown robust association with multiple psychiatric disorders, including schizophrenia (Ikeda et al., 2018), MD (Howard et al., 2019), alcohol dependence (Zhou et al., 2020), and various mental health measures, such as neuroticism (Nagel et al., 2020), well-being (Baselmans et al., 2019), and cigarette smoking (Liu et al., 2019). *DRD2* also interacts with medications for both psychiatric conditions and neuropathic pain (e.g., tiapride) (Gilron and Coderre, 2007).

*NRXN1* (*P*_meta_ = 1.62 × 10^–8^, *P*_OA_ = 3.78 × 10^–6^, *P*_MD_ = 5.54 × 10^–5^) is another well-known psychiatric risk gene that encodes neurexins, cell-surface receptors that act at the synapses to help regulate neurotransmission (Hu et al., 2019). Similar to *DRD2*, *NRXN1* interacts with a range of psychiatric medications such as duloxetine, which is used for MD, generalized anxiety disorder (GAD), fibromyalgia, chronic musculoskeletal pain, and osteoarthritis of the knee (Jenkins et al., 2014; Maciukiewicz et al., 2018). The *FOXP2* gene is another OA/MD risk gene we identified (*P*_meta_ = 1.07 × 10^–11^, *P*_OA_ = 1.14 × 10^–7^, *P*_MD_ = 1.96 × 10^–6^) and a member of the “mechanosensory behavior” gene set. This gene encodes the forkhead box protein P2, a transcription factor that is expressed in the brain, among other organs, and regulates the expression of a range of different genes (Becker et al., 2018). *FOXP2* has been implicated in attention deficit-hyperactivity disorder (Demontis et al., 2019), schizophrenia (Lam et al., 2019), and multisite chronic pain (Johnston et al., 2019). Similarly, *STRA6* is a shared OA/MD risk gene (*P*_meta_ = 1.20 × 10^–9^, *P*_OA_ = 2.96 × 10^–7^, *P*_MD_ = 5.35 × 10^–5^) and involved in “mechanosensory behavior.” This gene encodes a membrane protein that transports retinol across the cell membrane as part of vitamin A metabolism (Noy, 2016). The *STRA6* gene has been associated with behavioral inhibitory control (Weafer et al., 2017), as well as knee and hip osteoarthritis (Tachmazidou et al., 2019).

Third, although our cross-disorder meta-analysis did not find specific enrichment of genes involved in neuroinflammation, investigations of drug-gene interaction data suggest a potential biologic link relating OA and MD through the Hypothalamic-Pituitary-Adrenal (HPA) axis. Notably, *CRHR1*, the OA risk gene identified in the latest GWAS by Tachmazidou et al. (2019), plays a key role in the HPA axis. This gene is related to long-term depression and neuroactive ligand-receptor interaction in the brain (Schierloh et al., 2007), while interacting with 19 drugs indicated for MD and other mood disorders, anxiety disorders, and alcohol and drug dependence (Liu et al., 2007; Geng et al., 2014). The role of the HPA axis and implication of neuroinflammation in OA and psychosocial conditions, including anxiety and depression, has been well established (Loggia et al., 2015; Albrecht et al., 2018, 2019a,2019b). Carlesso et al. (2016) reported higher cortisol production in OA, which may be associated with deleterious effects on bone density and muscle mass (Sato et al., 2018). Similarly, elevated cortisol levels are associated with persistent depressive symptoms, with strong links to somatic symptoms like poor sleep and low energy (Iob et al., 2020). In fact, symptoms of cortisol dysfunction include fatigue, depression, and pain (Hannibal and Bishop, 2014), and patients with MD also frequently experience unexplained painful physical symptoms (UPPS) (Jaracz et al., 2016).

Lastly, we identified several OA-specific risk genes that interact with various psychiatric medications, suggesting the importance of integrative clinical care. The OA risk gene *CYP1A2* (*P*_OA_ = 2.47 × 10^–10^, *P*_MD_ = 0.782) encodes a cytochrome P450 superfamily enzyme that catalyzes reactions involved in the metabolism of drugs, chemicals, and various substrates (Kapelyukh et al., 2019). *CYP1A2* interacts with multiple psychiatric medications (e.g., clozapine, escitalopram, and paroxetine) indicated for MD, schizophrenia, anxiety disorders, and post-traumatic stress disorder (Kohlrausch et al., 2013; Kuo et al., 2013). Another OA risk gene *ITIH3* (*P*_OA_ = 2.59 × 10^–7^, *P*_MD_ = 5.27 × 10^–6^) interacts with antipsychotics clozapine (Brandl et al., 2016) and has been linked to depression (Amare et al., 2020), schizophrenia (Ikeda et al., 2018), and ASD (Xie et al., 2020), among other disorders. This gene has also shown genome-wide significant and independently replicated SNP-based associations with anxiety (Nagel et al., 2018) and risk-taking behavior (Karlsson Linnér et al., 2019). Our drug-gene interaction search also revealed a link between the OA-risk gene *SLC39A8* (*P*_OA_ = 2.14 × 10^–7^, *P*_MD_ = 0.080) and risk of developing alcoholism (Zhou et al., 2020). *SLC39A8* encodes a ubiquitously expressed metal cation transporter (Nebert and Liu, 2019). This gene exhibits extensive pleiotropy and is associated with a range of diseases including schizophrenia, Parkinson’s disease, and inflammatory bowel disease (Nebert and Liu, 2019). *DPEP1* is another OA risk gene (*P*_OA_ = 1.32 × 10^–8^, *P*_MD_ = 0.643), encoding an enzyme that hydrolyzes a range of substrates and participates in liver and lung neutrophil recruitment, interacts with glutamic acid, which may ameliorate symptoms of schizophrenia and help control alcoholism (Hammarström, 1983; Murphy and Gijón, 2007; Vance and Vance, 2008; Buczynski et al., 2009; Claesson, 2009; Choudhury et al., 2019). In addition, *DPEP1* has also shown associations with mathematical ability (Lee et al., 2018) and white matter microstructure (Zhao et al., 2019). Further investigation is warranted to decipher the functional role of OA-risk genes in the brain, as well as their interactions with psychiatric medications.

There are several limitations of the present study that we recognize. First, our bi-directional MR analysis indicates that there is no single directional causal effect between OA and MD. It is important to note that like all statistical methods, MR has key assumptions, such as no association between instrumental variables and any potential confounder (e.g., assortative mating). Within-family MR designs will be helpful to clarify issues related to potential uncontrolled confounders (Brumpton et al., 2020). Secondly, shared genetic effects can occur due to genuine pleiotropy, but there is also the possibility that LD among genetic loci or population stratifications (Haycock et al., 2016) may induce artificial or spurious pleiotropy. Robust genome-wide genetic correlations and inspection of our cross-disorder analysis data, however, overrule that these phenomena are the major factors driving the shared genetic relationships between OA and MD. Third, it is possible that comorbid cases of MD and OA may have contributed to pleiotropy we detected. This is a challenging issue for any large-scale genomic studies where comprehensive data on disorder comorbidity are not available. On the other hand, when multiple phenotypes overlap in their genetic risk architecture, we would expect increased comorbidity between patients with corresponding disorders. Thus, to the extent that comorbidity is related to genetic overlap, it is not confound but a reflection of the pleiotropy we are detecting. It will be important for future studies to investigate the impact of comorbidity in pleiotropy analysis. Fourth, stringent QC and standardization is necessary for cross-disorder analysis of two separately conducted GWAS summary statistics. We thus applied strict QC procedures that excluded about 5% of SNPs. This was necessary but may have resulted in the loss of power for subsequent gene and pathway-based analysis. OA and MD datasets also have different sample sizes, which may affect statistical power to detect pleiotropic risk loci. Fifth, both OA and MD have sex and age-dependent disease-prevalence and symptom severity. While we could not evaluate the relevance of sex and age in the shared genetic basis of the two disorders due to a lack of data, it is an important direction of our future research. Lastly, we analyzed samples of European ancestry, so our results may not generalize to other populations. In fact, the genetic architecture of OA and MD in other populations remains largely unexplored. Concerted efforts are warranted for collecting and interrogating non-European cohort samples, with ultimate aims of reducing the health disparity.

In summary, we have performed a cross-disorder genome-wide data analysis to elaborate the genetic relationships between OA and MD, two chronic and disabling conditions that affect millions of people worldwide. Our findings demonstrate that OA and MD share common genetic risk mechanisms, one of which centers on the neural response to the sensation of mechanical stimulus. Further investigation is warranted to elaborate the etiologic mechanisms of the mechanosensory behavior genes, as well as to develop effective strategies for early intervention and integrative clinical care of these serious health conditions.

## Data Availability Statement

Publicly available datasets were analyzed in this study. This data can be found here: https://www.med.unc.edu/pgc/download-results/ and https://www.ebi.ac.uk/gwas/.

## Author Contributions

PL conceived the project idea. PL, J-YJ, JS, ML, and MF developed the study. SB, J-YJ, NN, and MS ran the data analysis. All authors contributed to data interpretation, manuscript drafting and editing, and agreed to the submission of this manuscript.

## Conflict of Interest

MF has financial disclosure listed at: https://mghcme.org/app/uploads/2021/04/MF-Disclosures-Lifetime-updated-April-2021.pdf. The remaining authors declare that the research was conducted in the absence of any commercial or financial relationships that could be construed as a potential conflict of interest.

## Publisher’s Note

All claims expressed in this article are solely those of the authors and do not necessarily represent those of their affiliated organizations, or those of the publisher, the editors and the reviewers. Any product that may be evaluated in this article, or claim that may be made by its manufacturer, is not guaranteed or endorsed by the publisher.
