# Supplementary material for: Cross-Disorder Genomics Data Analysis Elucidates a Shared Genetic Basis Between Major Depression and Osteoarthritis Pain
Source: Front Genet. 2021 Sep 16;12:687687. doi: 10.3389/fgene.2021.687687 (PMC8481820; doi:10.3389/fgene.2021.687687)
Supplement: Supplementary file 1 [file Data_Sheet_1.PDF]

Supplemental Tables

Table S1. Statistical power calculations for different genetic correlations Standard error, non-centrality parameter of the chi-squared test statistic (NCP), and power calculated using GCTA-GREML Power Calculator for bivariate (OA/MD) and univariate (OA and MD) analyses. Statistical power was calculated for genetic correlations ranging from 0.1 to 0.5.

Table S2. Genomic risk loci and lead SNPs identified by FUMA Information about FUMA-identified genomic risk loci, including chromosome, position, p-value, and number and names of independent significant SNPs.

Table S3. Positional, eQTL, and Hi-C mapping of genes by FUMA Information about 1,524 genes mapped by FUMA using positional, eQTL, and Hi-C mapping data. Table includes chromosome, whether positional, eQTL, or chromatin interaction (Hi-C) mapping was performed, and maximum CADD score (used to identify deleteriousness of SNPs), among other gene information.

Table S4. General tissue expression of genes using FUMA Expression of 17,140 genes in 30 general tissue types using FUMA. Table includes beta, beta standard deviation (Beta SD), standard error (SE), and p-value.

Table S5. Specific tissue expression of genes using FUMA Expression of 17,140 genes in 54 specific tissue types using FUMA. Table includes beta, beta standard deviation (Beta SD), standard error (SE), and p-value.

Table S6. Top gene-set enrichment analysis results Results of gene-set enrichment analysis for top 20 gene sets. Table includes number of genes in the gene set, meta-analysis beta, standard deviation (SD), and standard error (SE) values, and p-values (OA, MD, meta-analysis, and Bonferroni-adjusted).

Table S7. OA/MD risk genes Information about 42 genes significantly associated with both OA and MD (gene-based $P_{meta} \leq 2.64\times10^{-6}$, $P_{OA} \leq 1\times10^{-4}$, $P_{MD} \leq 1\times10^{-4}$). Table includes chromosome, full gene name, and p-values for OA, MD, and meta-analysis, among other information.

Table S8. Cell-type specific RNA expression for shared OA/MD risk genes Results of brain-cell-type-specific expression analysis of 42 shared OA/MD risk genes. Table includes cell-type specific expression for 6 brain cell types as mean (-log10 Benjamini-Hochberg adjusted p-value of comparative analysis) and standard error (SE). A mean expression threshold of at least 5 and a Z-score (mean/SE) of 2 was used to designate significant expression.

Table S9. Brain-area expression for shared OA/MD risk genes Results of temporal brain expression analysis of shared OA/MD risk genes. Table includes whether genes show significant expression (log2-transformed signal intensity ≥ 6) in six brain regions (cerebellar cortex (CBC), mediodorsal nucleus of the thalamus (MD), striatum (STR), amygdala (AMY), hippocampus (HIP), and neocortex (NCX)) prenatally and postnatally, as well as the general spatiotemporal expression pattern.

Table S10. Full gene list for drug-gene interaction search Full list of OA risk, MD risk, shared risk, and “mechanosensory behavior” genes used to conduct our drug-gene interaction search.

Table S11. Additional information about relevant drug-gene interactions Drug-gene interaction information for 16 genes classified as OA, MD, shared, and mechanosensory. Table includes database sources, a full list of relevant drugs, their indications and associated conditions, and additional notes.

Table S1. Statistical power calculations for different genetic correlations

**Analysis Type**	**Genetic Correlation**	**Cases (MD)**	**Cases (OA)**	**Controls (MD)**	**Controls (OA)**	**Disease Risk (MD)**	**Disease Risk (OA)**	**Heritability (MD)**	**Heritability (OA)**	**Standard Error**	**NCP**	**Power**
Bivariate	0.1	170756	77052	329443	378169	0.15	0.0671	0.0796	0.0756	0.0090	123.562	1.0000
Bivariate	0.2	170756	77052	329443	378169	0.15	0.0671	0.0796	0.0756	0.0091	479.087	1.0000
Bivariate	0.3	170756	77052	329443	378169	0.15	0.0671	0.0796	0.0756	0.0094	1025.52	1.0000
Bivariate	0.4	170756	77052	329443	378169	0.15	0.0671	0.0796	0.0756	0.0097	1706.93	1.0000
Bivariate	0.5	170756	77052	329443	378169	0.15	0.0671	0.0796	0.0756	0.0101	2465.03	1.0000
Univariate	NA	170756	NA	329443	NA	0.15	NA	0.0796	NA	0.0008	8962.3	1.0000
Univariate	NA	NA	77052	NA	378169	NA	0.0671	NA	0.0756	0.0011	4350.11	1.0000

Table S4. General tissue expression of genes using FUMA

Tissue	# Genes	Beta	Beta SD	SE	P
Brain	17140	0.043668	0.075778	0.0086072	1.98E-07
Pituitary	17140	0.040811	0.076011	0.010333	3.94E-05
Nerve	17140	0.029855	0.060058	0.012923	0.010445
Ovary	17140	0.029381	0.059528	0.012916	0.011467
Cervix Uteri	17140	0.040578	0.079677	0.01803	0.012213
Muscle	17140	0.014184	0.026823	0.0087997	0.05351
Uterus	17140	0.023447	0.047755	0.015296	0.062662
Blood Vessel	17140	0.020961	0.04251	0.014825	0.078714
Testis	17140	0.0088424	0.015254	0.0070633	0.10532
Fallopian Tube	17140	0.0052623	0.010204	0.017536	0.38206
Colon	17140	0.0043094	0.0080616	0.019477	0.41245
Prostate	17140	0.0023283	0.0044142	0.017378	0.44671
Adrenal Gland	17140	0.00054697	0.0010549	0.012922	0.48312
Heart	17140	-0.0024065	-0.004137	0.012255	0.57784
Bladder	17140	-0.0049441	-0.009631	0.020144	0.59694
Skin	17140	-0.0033849	-0.006452	0.012687	0.60519
Breast	17140	-0.009019	-0.017334	0.019723	0.67626
Vagina	17140	-0.0076158	-0.01456	0.016073	0.68219
Adipose Tissue	17140	-0.010492	-0.020585	0.016751	0.73445
Esophagus	17140	-0.022343	-0.042067	0.02148	0.85086
Thyroid	17140	-0.014853	-0.029402	0.013698	0.86088
Blood	17140	-0.011564	-0.020719	0.0078355	0.92999
Lung	17140	-0.022461	-0.043404	0.013162	0.95603
Pancreas	17140	-0.021101	-0.035245	0.011202	0.97018
Stomach	17140	-0.037234	-0.067587	0.016994	0.98577
Liver	17140	-0.018262	-0.032858	0.0083112	0.98599
Kidney	17140	-0.027113	-0.047649	0.011834	0.98902
Spleen	17140	-0.025136	-0.049773	0.0098024	0.99483
Small Intestine	17140	-0.038429	-0.070623	0.013426	0.99789
Salivary Gland	17140	-0.043356	-0.080652	0.013699	0.99922

Table S5. Specific tissue expression of genes using FUMA

Tissue	# Genes	Beta	Beta SD	SE	P	P Bonferroni
Brain Cortex	17140	0.050654	0.092307	0.0088664	5.67E-09	3.05975E-07
Brain Cerebellum	17140	0.042329	0.084003	0.0078242	3.20E-08	1.72989E-06
Brain Frontal Cortex BA9	17140	0.046319	0.085303	0.0085832	3.45E-08	1.86543E-06
Brain Cerebellar Hemisphere	17140	0.038247	0.076742	0.0075946	2.41E-07	1.29902E-05
Brain Anterior cingulate cortex BA24	17140	0.044443	0.078449	0.0090432	4.50E-07	2.43103E-05
Brain Amygdala	17140	0.038814	0.066062	0.0098635	4.18E-05	0.002256228
Brain Nucleus accumbens basal ganglia	17140	0.037284	0.064637	0.0094799	4.22E-05	0.002276694
Brain Hippocampus	17140	0.036338	0.061468	0.010014	0.000143	0.00771714
Brain Caudate basal ganglia	17140	0.035393	0.061082	0.0098518	0.000164	0.00887166
Brain Hypothalamus	17140	0.034158	0.058734	0.010136	0.000377	0.02034126
Brain Putamen basal ganglia	17140	0.032723	0.05563	0.0099435	0.000501	0.02703024
Pituitary	17140	0.031861	0.05934	0.011148	0.002135	0.1152738
Brain Substantia nigra	17140	0.021219	0.036489	0.010737	0.024073	1
Cells Cultured fibroblasts	17140	0.008383	0.017959	0.0082088	0.15358	1
Brain Spinal cord cervical c-1	17140	0.010509	0.018869	0.010719	0.16344	1
Testis	17140	0.0047861	0.008256	0.0070586	0.24888	1
Muscle Skeletal	17140	0.0051717	0.00978	0.0087847	0.27803	1
Nerve Tibial	17140	0.0071672	0.014418	0.01247	0.28274	1
Artery Tibial	17140	0.0069958	0.014398	0.012465	0.28733	1
Ovary	17140	0.0066544	0.013482	0.012145	0.29189	1
Cervix Ectocervix	17140	0.0037683	0.007383	0.015291	0.40267	1
Colon Sigmoid	17140	-0.0009525	-0.00186	0.015623	0.52431	1
Esophagus Gastroesophageal Junction	17140	-0.0027692	-0.00545	0.015998	0.56871	1
Cervix Endocervix	17140	-0.0047962	-0.00949	0.014604	0.62869	1
Uterus	17140	-0.0063424	-0.01292	0.013774	0.67741	1
Esophagus Muscularis	17140	-0.010376	-0.02046	0.015464	0.74888	1
Adipose Subcutaneous	17140	-0.016571	-0.03305	0.013287	0.89382	1
Artery Aorta	17140	-0.016136	-0.03291	0.012695	0.89813	1
Heart Atrial Appendage	17140	-0.015578	-0.02794	0.012076	0.90145	1
Cells EBV-transformed lymphocytes	17140	-0.0094151	-0.02053	0.0061505	0.93708	1
Adrenal Gland	17140	-0.019139	-0.03691	0.012502	0.93709	1
Fallopian Tube	17140	-0.023903	-0.04635	0.014825	0.94654	1

Prostate	17140	-0.026145	-0.04957	0.015055	0.95876	1
Heart Left Ventricle	17140	-0.020388	-0.034	0.011587	0.96075	1
Whole Blood	17140	-0.014517	-0.02617	0.0070907	0.97968	1
Vagina	17140	-0.029578	-0.05655	0.013927	0.98315	1
Skin Sun Exposed Lower leg	17140	-0.02225	-0.04315	0.0097598	0.98868	1
Bladder	17140	-0.037134	-0.07234	0.016266	0.98877	1
Breast Mammary Tissue	17140	-0.036301	-0.06977	0.015651	0.98981	1
Artery Coronary	17140	-0.035211	-0.07005	0.014784	0.99138	1
Thyroid	17140	-0.030102	-0.05959	0.012165	0.99332	1
Skin Not Sun Exposed Suprapubic	17140	-0.024452	-0.04712	0.0097292	0.99401	1
Kidney Medulla	17140	-0.031976	-0.05907	0.01156	0.99716	1
Lung	17140	-0.03187	-0.06159	0.011336	0.99753	1
Pancreas	17140	-0.030809	-0.05146	0.010717	0.99798	1
Liver	17140	-0.023259	-0.04185	0.0080384	0.99809	1
Colon Transverse	17140	-0.044272	-0.08134	0.014327	0.999	1
Kidney Cortex	17140	-0.038712	-0.06794	0.011249	0.99971	1
Spleen	17140	-0.031395	-0.06217	0.009128	0.99971	1
Adipose Visceral Omentum	17140	-0.0483	-0.09375	0.013833	0.99976	1
Stomach	17140	-0.054911	-0.09968	0.014936	0.99988	1
Small Intestine Terminal Ileum	17140	-0.046349	-0.08518	0.011928	0.99995	1
Esophagus Mucosa	17140	-0.038004	-0.07343	0.0096418	0.99996	1
Minor Salivary Gland	17140	-0.050759	-0.09442	0.012098	0.99999	1

Table S6. Top gene-set enrichment analysis results

Gene set	# Genes	Beta Meta-Analysis	Beta SD	SE	P OA	P MD	P Meta-Analysis	P Bonferroni
Mechanosensory behavior	15	1.431	0.04	0.262	1.76E-05	2.5E-06	2.45E-08	0.000254
Intrinsic component of postsynaptic density membrane	54	0.63855	0.034	0.145	0.31324	4.4E-10	5.08E-06	0.052556
Response to auditory stimulus	23	0.9388	0.033	0.224	0.005299	0.00192	1.4E-05	0.14469
Observational learning	8	1.3396	0.028	0.327	7.49E-08	0.00401	2.13E-05	0.22058
Learned vocalization behavior or vocal learning	10	1.2956	0.03	0.317	0.00011	0.0013	2.17E-05	0.224587
Postsynaptic density membrane	73	0.51329	0.032	0.127	0.5853	2E-08	2.55E-05	0.264235
Dawson methylated in lymphoma tcl1	56	0.62746	0.034	0.157	0.15013	5E-05	3.37E-05	0.348717
Branching morphogenesis of a nerve	10	1.4643	0.034	0.376	0.18113	1.2E-08	4.97E-05	0.514287
Positive regulation of skeletal muscle cell differentiation	6	1.735	0.031	0.465	1.7E-05	0.00222	9.47E-05	0.980666
Dynactin binding	12	1.184	0.03	0.317	0.018032	0.0044	9.54E-05	0.987757
Neuron spine	154	0.32176	0.029	0.087	0.013641	1E-05	0.000111	1
Intrinsic component of presynaptic membrane	83	0.45643	0.03	0.124	0.035438	3.3E-06	0.000115	1
Peristalsis	10	1.329	0.031	0.362	0.049799	0.18107	0.00012	1

Browne hcmv infection 20hr dn	97	0.41747	0.03	0.114	0.000214	0.03948	0.000121	1
Chondrocyte development	47	0.58284	0.029	0.159	7.38E-05	0.5052	0.000127	1
Regulation of mesenchymal stem cell differentiation	6	1.5666	0.028	0.431	0.003852	0.00947	0.000137	1
Positive regulation of protein localization to centrosome	7	1.287	0.025	0.355	0.001416	0.12595	0.000144	1
Regulation of protein localization to centrosome	10	1.0719	0.025	0.298	0.005364	0.19098	0.000163	1
Negative regulation of axonogenesis	63	0.46256	0.027	0.132	0.032878	0.00527	0.000222	1
Anterograde dendritic transport	5	1.5894	0.026	0.457	0.32988	0.11904	0.000254	1
